# Occurrence of hepatitis B and C virus infection in socioeconomic population strata from Recife, Pernambuco, Northeast Brazil

**DOI:** 10.1590/1980-549720240033

**Published:** 2024-07-01

**Authors:** Carolline de Araújo Mariz, Cynthia Braga, Maria de Fátima Pessoa Militão de Albuquerque, Carlos Feitosa Luna, Daniela Medeiros Salustiano, Naishe Matos Freire, Clarice Neuenschwander Lins de Morais, Edmundo Pessoa Lopes

**Affiliations:** I Fundação Oswaldo Cruz, Instituto Aggeu Magalhães – Recife (PE), Brazil; II Faculdade de Medicina de Olinda – Olinda (PE), Brazil.; III Laboratório Central de Saúde Pública Dr. Milton Bezerra Sobral – Recife (PE), Brazil.; IV Universidade Federal de Pernambuco, Faculty of Medicine of Recife – Recife (PE), Brazil.

**Keywords:** Hepatitis B, Hepatitis C, Viral hepatitis, Socioeconomic status

## Abstract

**Objective:**

To estimate the probability of infection with hepatitis B (HBV) and C (HCV) viruses in different socioeconomic strata of the population of Recife, Northeast Brazil.

**Methods:**

Study carried out from samples obtained in a survey of residents of a large urban center that had a population base and stratified sampling with random selection of households using the “Brazil Sample” package in the R software. HBV (HBsAg) and anti-HCV was performed using immunochromatographic tests. In cases positive for HBsAg, anti-HBc and HBeAg were tested using chemiluminescence, as well as HBV-DNA using real-time PCR. For cases positive for anti-HCV, the search for this antibody was repeated by chemiluminescence and for HCV-RNA by real-time PCR. The occurrence of HBsAg and anti-HCV cases in the general population was estimated based on a theoretical negative binomial distribution.

**Results:**

Among 2,070 samples examined, 5 (0.24%) were HBsAg and 2 (0.1%) anti-HCV positive. The majority of cases had self-reported skin color as black/brown (6/7), education level up to high school (6/7), a steady partner (5/7) and lived in an area of low socioeconomic status (5/7).

**Conclusion:**

The occurrence of HBsAg and anti-HCV was lower than those previously found in population-based studies and slightly lower than the most recent estimates. Individuals with lower socioeconomic status should be a priority target of public health policies.

## INTRODUCTION

Viral hepatitis B and C represent significant public health challenges worldwide, with tens of millions of individuals estimated to be infected by each of these pathogens. These diseases contribute substantially to morbidity and mortality globally, primarily through the development of cirrhosis and hepatocellular carcinoma^1,2^.

In recent years, the incidence of HBV infection has shown a decline, likely attributed to the expanded availability of immunization and antiviral therapy since the 1990s^3^. Similarly, the incidence of HCV infection has also seen a decrease due to increased access to diagnostic tests and the introduction of new direct-acting antivirals over the past decade^4,5^. However, the endemicity levels of both viral infections continue to vary across regions of the globe, with higher rates observed in less developed countries and among low-income populations, particularly in Asia and Africa^5-7^. Globally, lower socioeconomic status has been associated with increased exposure to HBV and HCV infections^5,8-10^.

Brazil is presently categorized as a country with low endemicity for HBV and HCV infections. Moreover, it is listed among the signatory countries of the World Health Organization (WHO) objectives for the elimination of viral hepatitis by the year 2030^1,4-5,11^. However, despite this classification, Brazil remains a nation marked by significant social inequality^12^.

Research on the prevalence of viral hepatitis B and C conducted in Brazil is limited^13,14^, and the majority focuses on specific population subsets, including blood donors, hemodialysis patients, individuals in correctional facilities, or those experiencing poverty^8,15,16^. Additionally, there is a lack of recent literature examining the seroprevalence of viral hepatitis B and C on a population-wide scale, categorized according to the socioeconomic strata of the Brazilian population.

A serum sample obtained from an arbovirus seroprevalence survey conducted across various socioeconomic strata of the population in the city of Recife, Northeast Brazil, was utilized to estimate the likelihood of hepatitis B and C virus infections among participants in this survey.

## METHODS

This study utilized samples obtained from a previous population-based study^17^ conducted with stratified sampling and random selection of units (households) using the “Amostra Brasil” package in the R software^18^.

The population survey was conducted on a sample of residents of Recife, aged between 5 and 65 years, from September 2018 to February 2019^17^.

The municipality of Recife has an estimated population of 1,645,727 inhabitants and covers a territorial area of 218,843 km². It is divided into 94 neighborhoods distributed across six political-administrative regions^19^. Recife is situated in the third most densely populated metropolitan area in the country and is recognized as the Brazilian capital with the highest level of social inequality, as indicated by the last three demographic censuses^20^. Despite spatial segregation of social classes in certain areas, poverty and affluence often coexist closely within the same urban space. Approximately 40% of families in the municipality have a *per capita* monthly income of up to ½ the minimum wage, equivalent to approximately 136.4 US dollars, signifying a situation of social vulnerability for a significant portion of the population^19^.

In brief, the survey sampling was stratified into three socioeconomic levels (high, intermediate, and low) and conducted by conglomerate, with selection in two stages. Firstly, census tracts were randomly chosen at each socioeconomic level (first stage), followed by the random selection of households within the selected tracts (second stage).

All residents within the selected households, falling within the study age range, were invited to participate. Upon reading and signing the Informed Consent, individual and household information was collected through a standardized questionnaire administered via interview. Subsequently, venous blood samples of 10 mL for adults and 5 mL for children were obtained for serological testing. The blood samples were collected in vacuum collection tubes containing clot-activating separating gel and stored at the Virology and Experimental Therapy Laboratory, Instituto Aggeu Magalhães (IAM), Oswaldo Cruz Foundation (Fiocruz), where they were processed and deep-frozen at -80°C.

The investigation of viral hepatitis B and C was conducted using serum samples obtained from all participants in the arbovirus survey. For the detection of HBV surface antigen (HBsAg), the Bioclin brand kit was utilized, employing an immunochromatographic method for rapid and qualitative determination. Additionally, the Abon brand rapid test, utilizing lateral flow immunochromatography, was employed for the qualitative detection of anti-HCV antibody. In cases positive for HBsAg, further testing for anti-HBc and HBeAg was performed using chemiluminescence (Architect, Abbott), along with HBV-DNA detection using real-time PCR (Alinity TM m, Abbott). Similarly, for cases positive for anti-HCV, the search for this antibody was repeated via chemiluminescence (Architect, Abbott), and HCV-RNA detection was conducted using real-time PCR (Alinity TM m, Abbott).

The following exposure variables were evaluated:

A) related to the individual — age (categorized into two groups: ≤40 years and >40 years), gender, self-reported skin color (white, black, and brown), sexual practice with a steady partner (yes and no), and blood transfusion (yes and no);
B) related to the household — socioeconomic stratum based on information on the percentage of household heads with income below two minimum wages, including those without income, using data from the 2010 Demographic Census^20^ (categorized as: high, intermediate, and low), number of residents per household (categorized as: up to 3 residents, between 3 and 5 residents, and >5 residents), type of household (apartment and house), gender of the head of the family, income in minimum wages of the head of the family (≤2 and >2 minimum wages), and level of complete education with approval of the head of the family (elementary, high school, and higher education).

Data management was conducted using the REDCap electronic platform, hosted at IAM, Fiocruz-PE, and data analysis was performed using the Stata program, version 15, and the R software, version 4.0.2. Descriptive analysis was carried out, and the distribution of variables was presented through tables. Pearson’s Chi-square test with Rao-Scott correction^21^ was utilized to compare the characteristics of the selected households and between socioeconomic strata. A negative binomial distribution^22^ was employed to estimate the probability of the occurrence of a given event, HBsAg, and anti-HCV, given the occurrence of an observed number of “successes” in the repetition of “n” experiments (examined). It should be noted that the intention was not to estimate parameters such as prevalence involving rare events; the estimation of probabilities would be free from sampling bias. This study complied with the ethical principles of Resolution no. 466, dated December 12, 2012, of the National Health Council of Brazil, and was approved by the CEP/Conep system under CAAE no. 51880021.0.0000.8807.

## RESULTS

The survey data indicate that among the 892 households studied, a total of 2,070 individuals were assessed. Of these, 416 individuals (20.1%) belonged to the high socioeconomic stratum, 726 (35.1%) to the intermediate stratum, and 928 (44.8%) to the low socioeconomic stratum. The average number of residents per household was found to be 1.61 (±0.03).

The majority of visited households were houses (74.5%), with up to three residents (56.7%). A higher percentage of households with five or more residents was observed in the low socioeconomic stratum (15.1%). Family heads from the high socioeconomic stratum exhibited higher levels of education (67.5%) and income (74.2%) compared to those from the intermediate and low socioeconomic strata, respectively (Table 1).

**Table 1:** Characteristics of selected households according to socioeconomic strata in Recife (PE), Brazil, 2019.

Characteristics	Total n (%)	Socioeconomic stratum			p value*
		High	Intermediary	Low	
Studied households	892 (100)	231 (25.9)	349 (39.1)	312 (35.0)	-
Number of residents per household					
Up to 3	506 (56.7)	140 (60.6)	211 (60.5)	155 (49.7)	0.001
>3–5	299 (33.5)	78 (33.8)	111 (31.8)	110 (35.2)	
>5	87 (9.8)	13 (5.6)	27 (7.7)	47 (15.1)	
Type of household					
House	664 (74.5)	84 (36.4)	280 (80.5)	300 (96.2)	<0.001
Apartment	227 (25.5)	147 (63.6)	68 (19.5)	12 (3.8)	
Head of the household characteristics					
Gender					
Female	435 (48.8)	118 (51.1)	172 (49.3)	145 (46.5)	0.552
Male	457 (51.2)	113 (48.9)	177 (50.7)	167 (53.5)	
Income in minimum wages					
≤2	489 (57.7)	57 (25.8)	188 (56.8)	244 (82.4)	<0.001
>2	359 (42.3)	164 (74.2)	143 (43.2)	52 (17.6)	
Education level					
Elementary	292 (34.3)	32 (14.0)	115 (34.2)	145 (50.5)	<0.001
High School	293 (34.4)	42 (18.4)	125 (37.2)	126 (43.9)	
Higher Education	266 (31.3)	154(67.6)	96 (28.6)	16 (5.6)	

* Pearson’s χ^2^ test with Rao-Scott correction^22^.

**Figure 1:**
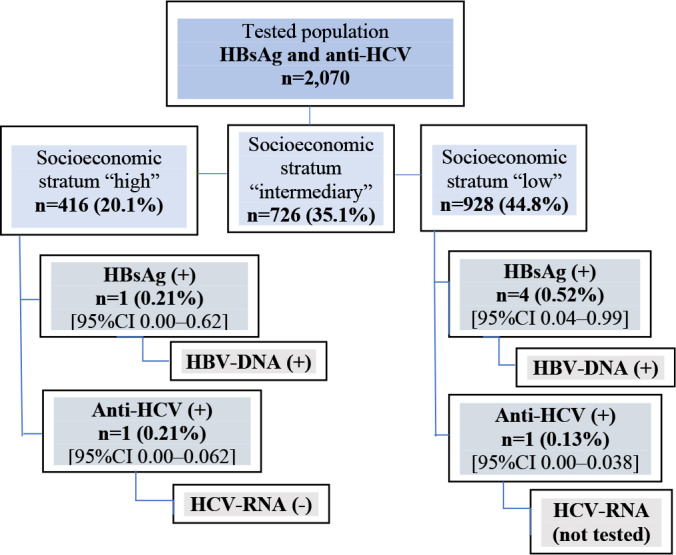
Flowchart of HBsAg and anti-HCV testing by socioeconomic stratum in Recife (PE), Brazil, 2019.

The distribution of markers according to socioeconomic stratum is depicted in Figure 1. Out of the total 2,070 samples analyzed, five (0.24%) tested positive for HBsAg, and two (0.1%) were positive for anti-HCV. Notably, no more than one case was detected per household. Regarding the occurrence of two cases of anti-HCV in 2,070 samples analyzed, the probability of such cases is less than 1.5 x 10^4^ (p=0.039). Similarly, for HBsAg, the five positive cases indicate a probability of occurrence of less than 1 x 10^3^ (p=0.059).

The occurrence of viral hepatitis B and C, categorized by gender and age range, along with their respective confidence intervals, are presented in Table 2. Among those positive for HBsAg or anti-HCV, the majority were identified as black or brown (6/7), had an education level of up to high school (6/7), had a partner (5/7), and were from a low socioeconomic stratum (5/7). Notably, none of the seven positive cases reported a history of blood transfusion or receipt of blood products.

**Table 2:** Occurrence of hepatitis B and C viral infection according to gender and age range. Recife (PE), Brazil, 2019.

	Examined	Hepatitis B		Hepatitis C	
		HBsAg		Anti-HCV	
		Pos	Prev (95%CI)	Pos	Prev (95%CI)
Total	2,070	5	0.24 (0.04–0.50)	2	0.10 (0.00–0.24)
Gender					
Female	1,212	3	0.27 (0.00–0.57)	1	0.10 (0.00–0.29)
Male	858	2	0.19 (0.00–0.66)	1	0.10 (0.00–0.30)
Age range (in years)					
≤40	1,173	4	0.41 (0.02–0.79)	1	0.10 (0.00–0.30)
>40	897	1	0.10 (0.00–0.29)	1	0.10 (0.00–0.29)

Pos: positive; Prev: prevalence; 95%CI: 95% confidence interval.

The results of the HBeAg and HBV-DNA testing for the five identified cases are presented in Table 3. Regarding the two cases positive for anti-HCV, one individual exhibited an undetectable HCV-RNA test result (indicative of previous antiviral treatment), while the biological sample from the other case was insufficient for viral RNA research.

**Table 3:** Demographic characteristics and results of HBeAg and viral load of the five patients with positive HBsAg. Recife (PE), Brazil, 2019.

Case	Gender	Age (years)	Socioeconomic stratum	HBV-DNA		HBeAg
				UI/mL	Log	
1	F	39	Low	2,458	3.39	neg
2	M	38	Low	65	1.82	neg
3	M	36	Low	230,303,427	8.36	pos
4	F	56	High	8,008,840	6.90	pos
5*	F	38	Low	12	1.08	pos

* On antiviral treatment. F: female; M: male; Log: logarithm; pos: positive; neg: negative.

## DISCUSSION

The occurrence of HBsAg and anti-HCV in this study was found to be lower compared to rates reported in other Brazilian population-based studies^13,23^. It was noted that most positive cases resided in the low socioeconomic stratum, where the monthly income of the head of the family was less than two minimum wages.

The first population-based survey on the prevalence of viral hepatitis A, B, and C at a national level was conducted between 2005 and 2009, revealing a global prevalence of less than 1% for HBV and 1.38% for HCV. This survey encompassed of all capitals in each macro-region of the country, including the Federal District^13,14^. In the Northeast region specifically, a prevalence of 5.5% for HBV^13^ was observed, approximately 20 times higher than the prevalence found in the present study (0.24%). Additionally, a prevalence of 0.68% for HCV^14^ was reported, which is very close, albeit slightly higher than the prevalence found in our population (0.10%).

Other population-based prevalence studies of viral hepatitis have been conducted in Brazil. A study conducted in a large metropolis in the Southeast region in 1996 found a prevalence of HBsAg of 1.04% and anti-HCV of 1.42%^23^. Subsequently, in 1999, another population-based study carried out in a small city in the semi-arid region of Bahia found a prevalence of HBsAg of 2.6% and anti-HCV of 0.4%^24^. Fourteen years after the last national survey^13,14^, our results, evaluating 2,070 individuals from the capital of Pernambuco, indicate a reduction in the occurrence of viral hepatitis B and C in recent years. Undoubtedly, this decline can be attributed to the introduction of HBV vaccination in the national vaccination calendar and improvements in case management, including the availability of antiviral therapies such as nucleoside/nucleotide analogues and direct-acting antivirals against HBV and HCV, respectively^3,25^. These measures have made significant contributions to reducing the incidence of these infections, particularly in industrialized countries.

Corroborating findings from national and international prevalence studies, which have demonstrated the association of socioeconomic factors such as low levels of education and family income^8,26-28^, most cases identified in this study were in the low socioeconomic stratum and had lower levels of education. Recent estimates indicate that the majority of HBV and HCV infections are concentrated in countries with precarious socioeconomic conditions^5^, where access to education and healthcare, including vaccines and medications, is severely limited, thereby allowing for the persistence of transmission.

In this study, no statistically significant difference was observed between the occurrence of both markers and the age of the participants, consistent with previous studies^13,14^. Regarding gender, similar findings were observed in relation to the occurrence of viral markers B and C. Our results align with the study conducted across all Brazilian macro-regions between 2005 and 2009, which found no significant association between gender and hepatitis C antibodies in the population^14^. However, our findings differ from the study conducted in three regions of Brazil between 2004 and 2005, which reported a positive association between males and HBV infection in all regions studied^13^.

It is noteworthy that among the five patients with positive HBsAg, two exhibited very high viremia, and in both cases, HBeAg was also positive. All patients with positive markers were advised to seek specialized medical services, including the Hospital das Clínicas of Universidade Federal de Pernambuco.

The tracking of infection markers for HBV and HCV in a serum sample from a population, the size of which was calculated to estimate the seroprevalence of arboviruses, represents a limitation of this study. However, considering the availability of the serum bank based on a random sample of the general population, which is assumed to be healthy *a priori*, we do not believe there are any biases that would compromise the validity of the study.

It is noteworthy that prevalence studies involving viral hepatitis B and C in healthy individuals are rare, old, and often have smaller sample sizes compared to ours, with very few exceptions. For instance, a study conducted in the late 1990s to assess the prevalence of viral hepatitis A, B, C, and E in the city of São Paulo, with an estimated population of 9.6 million inhabitants at the time, randomly included 1,059 serum samples^23^. Generally, the most recent studies reporting higher prevalence of these markers were conducted in specific population groups at higher risk of infection, such as sex workers^29^, among others^15,16^.

The exclusion of individuals over 65 years of age represented another limitation, given the association between HCV infection and advancing age^30^. However, despite this exclusion criterion, no statistically significant differences were observed in the occurrence of both markers concerning age.

In summary, the findings of this study indicate a reduction in the occurrence of viral hepatitis B and C in a large urban center in the Northeast region, reflecting the current situation in the area. However, our results underscore the importance of intensifying epidemiological surveillance and enhancing the healthcare network aimed at addressing cases of HBV and HCV among populations with lower socioeconomic conditions. Following a nosological diagnosis, individuals should be referred for reevaluation and potential treatment, available in specialized services, to work toward the WHO’s goal of eliminating these viruses in the country by 2030^11^.
