# Supplementary material for: Occurrence of hepatitis B and C virus infection in socioeconomic population strata from Recife, Pernambuco, Northeast Brazil
Source: Rev Bras Epidemiol. 2024 Jul 1;27:e240033. doi: 10.1590/1980-549720240033 (PMC11221483; doi:10.1590/1980-549720240033)
Supplement: Translation

# Translation: Ocorrência da infecção pelo vírus das hepatites B e C em estratos socioeconômicos populacionais do município de Recife, Pernambuco, Nordeste do Brasil

**DOI:** 10.1590/1980-549720240033.2

**Authors:** Carolline de Araújo Mariz, Cynthia Braga, Maria de Fátima Pessoa Militão de Albuquerque, Carlos Feitosa Luna, Daniela Medeiros Salustiano, Naishe Matos Freire, Clarice Neuenschwander Lins de Morais, Edmundo Pessoa Lopes

**Affiliations:** I Fundação Oswaldo Cruz, Instituto Aggeu Magalhães – Recife (PE), Brasil; II Faculdade de Medicina de Olinda – Olinda (PE), Brasil.; III Laboratório Central de Saúde Pública Dr. Milton Bezerra Sobral – Recife (PE), Brasil.; IV Universidade Federal de Pernambuco, Faculdade de Medicina do Recife – Recife (PE), Brasil.

**Keywords:** Hepatite B, Hepatite C, Hepatites virais, Estrato socioeconômico

## Abstract

**Objetivo:**

Estimar a probabilidade da ocorrência de infecção pelos vírus das hepatites B (HBV) e C (HCV) em diferentes estratos socioeconômicos da população de Recife, Nordeste do Brasil.

**Métodos:**

Estudo realizado com base em amostras obtidas em um inquérito de residentes de um grande centro urbano que teve base populacional e amostragem estratificada com seleção aleatória dos domicílios por meio do pacote “Amostra Brasil” no *software* R. A pesquisa do antígeno de superfície do HBV (HBsAg) e do anti-HCV foi realizada por testes imunocromatográficos. Nos casos positivos para HBsAg, foram realizadas pesquisas do anti-HBc e do HBeAg por quimioluminescência, bem como do HBV-DNA, por meio de PCR em tempo real. Para os casos positivos para anti-HCV, foi repetida a pesquisa desse anticorpo por quimioluminescência e do HCV-RNA por PCR em tempo real. A ocorrência de casos HBsAg e anti-HCV na população geral foi estimada com base em uma distribuição teórica binomial negativa.

**Resultados:**

Dentre 2.070 amostras examinadas, cinco (0,24%) foram HBsAg e duas (0,1%) anti-HCV positivas. A maioria dos casos tinha cor de pele autorreferida como preta/parda (6/7), nível de escolaridade até o ensino médio (6/7), companheiro fixo (5/7) e morava em área de baixo estrato socioeconômico (5/7).

**Conclusão:**

A ocorrência de HBsAg e anti-HCV foi inferior às anteriormente encontradas em estudos de base populacional e pouco menor do que as estimativas mais recentes. Indivíduos que apresentam menor condição socioeconômica devem ser alvo prioritário das políticas públicas de saúde.

## INTRODUÇÃO

As hepatites virais B e C constituem um dos principais problemas de saúde pública no mundo, com estimativa de dezenas de milhões de infectados por cada um desses agentes. Essas enfermidades proporcionam elevada morbidade e mortalidade por cirrose e carcinoma hepatocelular em todo o planeta^1,2^.

A incidência da infecção pelo HBV vem diminuindo nos últimos anos, possivelmente devido à ampliação da oferta de imunização e da terapia antiviral a partir da década de 1990^3^. Já a incidência da infecção pelo HCV vem diminuindo em virtude da maior disponibilidade dos testes para diagnóstico e do tratamento com os novos antivirais de ação direta, na última década^4,5^. Todavia o nível de endemicidade para ambas as infecções virais ainda difere nas regiões do planeta, sendo maior entre os países menos desenvolvidos e nas populações de baixa renda, sobretudo da Ásia e África^5-7^. Em todo o mundo, o menor nível socioeconômico tem sido relacionado a uma maior exposição às infecções pelo HBV e HCV^5,8-10^.

O Brasil, atualmente, é classificado como um país de baixa endemicidade para as infecções pelos HBV e HCV, como também compõe a lista de países signatários das metas da Organização Mundial da Saúde (OMS) para eliminação das hepatites virais até o ano de 2030^1,4-5,11^, embora se configure como um país com acentuada iniquidade social^12^.

Estudos de prevalência das hepatites virais B e C conduzidos no Brasil são escassos^13,14^ e a maioria aborda grupos populacionais específicos, a exemplo de doadores de sangue, pacientes em hemodiálise, pessoas privadas de liberdade ou em situação de pobreza^8,15,16^. Além disso, não se encontram na literatura recente estudos sobre a soroprevalência das hepatites virais B e C de base populacional, de acordo com os estratos socioeconômicos da população brasileira.

Uma soroteca, proveniente de um inquérito de soroprevalência de arboviroses conduzido em diferentes estratos socioeconômicos populacionais na cidade do Recife, Nordeste do Brasil, foi utilizada para estimar a probabilidade da ocorrência de infecção pelos vírus das hepatites B e C entre os participantes deste inquérito.

## MÉTODOS

Trata-se de um estudo realizado a partir de amostras obtidas em estudo anterior^17^, que teve base populacional e amostragem estratificada com seleção aleatória das unidades (domicílios) por meio do pacote “Amostra Brasil” no *software* R^18^.

O inquérito populacional foi realizado em uma amostra de residentes do Recife, com idade entre 5 e 65 anos, no período de setembro de 2018 a fevereiro de 2019^17^.

O município de Recife tem população estimada de 1.645.727 habitantes e uma área territorial de 218.843 km², dividida em 94 bairros distribuídos em seis regiões político-administrativas^19^. A cidade está situada na terceira área metropolitana mais densamente povoada do país e é considerada a capital brasileira com maior índice de desigualdade social, segundo os últimos três censos demográficos^20^. Embora haja segregação espacial de classes sociais em algumas áreas, pobreza e riqueza coexistem proximamente no mesmo espaço da cidade. Cerca de 40% das famílias do município possuem rendimento mensal *per capita* de até ½ salário mínimo, o que equivale a aproximadamente 136,4 dólares americanos, caracterizando situação de vulnerabilidade social de relevante parcela da população^19^.

Sumariamente, a amostragem do inquérito, estratificada em três níveis socioeconômicos (alto, intermediário e baixo), foi por conglomerado, com seleção em dois estágios. Inicialmente, foi realizado o sorteio dos setores censitários em cada nível socioeconômico (primeiro estágio) e, em seguida, foi feita a seleção aleatória dos domicílios nos setores sorteados (segundo estágio).

Todos os moradores dos domicílios sorteados, na faixa etária do estudo, foram convidados a participar. Após a leitura e assinatura do Termo de Consentimento/Assentimento Livre e Esclarecido, informações individuais e relativas aos domicílios foram obtidas por meio de entrevista utilizando questionário padronizado. Em seguida, obteve-se uma amostra de sangue venoso de 10 mL nos adultos e 5 mL nas crianças para a realização dos testes sorológicos. As amostras de sangue foram acondicionadas em tubos de coleta à vácuo com gel separador ativador de coágulo e armazenadas no Laboratório de Virologia e Terapia Experimental, do Instituto Aggeu Magalhães (IAM), Fundação Oswaldo Cruz (Fiocruz), onde foram processadas e congeladas em *deep-freezer* a -80° C.

A pesquisa das hepatites virais B e C foi realizada com base nas amostras de soro de todos os participantes do inquérito de arboviroses. Utilizou-se o *kit* HBsAg, da marca Bioclin, por método imunocromatográfico para determinação rápida e qualitativa de antígeno de superfície do HBV, e o teste rápido da marca Abon, por imunocromatografia de fluxo lateral, que permite a detecção qualitativa do anticorpo anti-HCV. Nos casos positivos para o HBsAg, foram realizadas as pesquisas do anti-HBc e do HBeAg por quimioluminescência (Architect, Abbott), bem como do HBV-DNA, por meio de PCR em tempo real (Alinity TM m, Abbott). Para os casos positivos para o anti-HCV, foi repetida a pesquisa deste anticorpo por quimioluminescência (Architect, Abbott) e do HCV-RNA por PCR em tempo real (Alinity TM m, Abbott).

Foram avaliadas as seguintes variáveis de exposição:

A) relacionadas ao indivíduo — idade (categorizada em dois grupos: ≤40 anos e >40 anos), sexo, cor da pele autorreferida (branca, preta e parda), prática sexual com parceiro fixo (sim e não) e transfusão de sangue (sim e não);
B) relacionadas ao domicílio — estrato socioeconômico com base na informação sobre o percentual de chefes de família com renda inferior a dois salários mínimos, incluindo os sem renda, utilizando dados do Censo Demográfico de 2010^20^ (categorizado em: alto, intermediário e baixo), número de moradores por domicílio (categorizados em: até 3 moradores, entre 3 e 5 moradores e >5 moradores), tipo de domicílio (apartamento e casa), sexo do chefe da família, renda em salários mínimos do chefe de família (≤2 e >2 salários mínimos) e nível de escolaridade completo com aprovação do chefe de família (ensino fundamental, médio e superior).

O gerenciamento dos dados foi realizado utilizando a plataforma eletrônica *REDCap*, hospedada no IAM, Fiocruz-PE, e a análise dos dados foi realizada utilizando o programa *Stata*, versão 15 e o *software* R v. 4.0.2. Foi realizada a análise descritiva e a apresentação da distribuição das variáveis através de tabelas. Empregou-se o teste Qui-quadrado de Pearson com correção de Rao-Scott^21^ para comparar as características dos domicílios selecionados e entre os estratos socioeconômicos. Utilizou-se uma distribuição binomial negativa^22^ destinada a estimar a probabilidade de ocorrência de um determinado evento, HBsAg e anti-HCV, dada a ocorrência de um observado número de “sucessos” na repetição de “n” experimentos (examinados). Ressaltando-se que não se pretendeu estimar parâmetros como prevalência envolvendo eventos raros, a estimação de probabilidades estaria livre de vieses amostrais. Este estudo obedeceu aos preceitos éticos da Resolução n° 466, de 12 de dezembro de 2012, do Conselho Nacional de Saúde do Brasil, e foi aprovado pelo sistema CEP/Conep sob o CAAE n° 51880021.0.0000.8807.

## RESULTADOS

Dados do inquérito mostram que, entre os 892 domicílios estudados, um total de 2.070 indivíduos foram avaliados, dos quais 416 (20,1%) pertenciam ao estrato socioeconômico alto, 726 (35,1%) ao estrato intermediário e 928 (44,8%) ao estrato socioeconômico baixo. Foi verificado um número médio de moradores por dormitório de 1,61 (±0,03).

A maioria dos domicílios visitados era do tipo casa (74,5%) e tinha até três moradores (56,7%). Foi observado percentual maior de domicílios com cinco moradores ou mais no estrato socioeconômico baixo (15,1%). Os chefes de família do estrato alto tiveram maior escolaridade (67,5%) e renda (74,2%) quando comparados aos dos estratos socioeconômicos intermediário e baixo, respectivamente (Tabela 1).

**Tabela 1:** Características dos domicílios selecionados segundo estratos socioeconômicos em Recife (PE), Brasil, 2019.

Características	Total n (%)	Estrato socioeconômico			p-valor*
		Alto	Intermediário	Baixo	
Domicílios estudados	892 (100)	231 (25,9)	349 (39,1)	312 (35,0)	-
N° de moradores por domicílio					
Até 3	506 (56,7)	140 (60,6)	211 (60,5)	155 (49,7)	0,001
>3–5	299 (33,5)	78 (33,8)	111 (31,8)	110 (35,2)	
>5	87 (9,8)	13 (5,6)	27 (7,7)	47 (15,1)	
Tipo de domicílio					
Casa	664 (74,5)	84 (36,4)	280 (80,5)	300 (96,2)	<0,001
Apartamento	227 (25,5)	147 (63,6)	68 (19,5)	12 (3,8)	
Características do chefe da família					
Sexo					
Feminino	435 (48,8)	118 (51,1)	172 (49,3)	145 (46,5)	0,552
Masculino	457 (51,2)	113 (48,9)	177 (50,7)	167 (53,5)	
Renda em salários mínimos					
≤2	489 (57,7)	57 (25,8)	188 (56,8)	244 (82,4)	<0,001
>2	359 (42,3)	164 (74,2)	143 (43,2)	52 (17,6)	
Nível de escolaridade					
Fundamental	292 (34,3)	32 (14,0)	115 (34,2)	145 (50,5)	<0,001
Médio	293 (34,4)	42 (18,4)	125 (37,2)	126 (43,9)	
Superior	266 (31,3)	154(67,6)	96 (28,6)	16 (5,6)	

* Teste χ^2^ de Pearson com correção de Rao-Scott^22^.

A distribuição dos marcadores de acordo com estrato socioeconômico é apresentada na Figura 1. Do total de 2.070 amostras analisadas, cinco (0,24%) foram HBsAg e duas (0,1%) foram anti-HCV positivas. Não foi detectado mais de um caso por domicílio. Dado a ocorrência de dois casos do anti-HCV em 2.070 amostras analisadas, a probabilidade de ocorrência de casos é menor de 1,5 x 10^4^ (p=0,039). Em relação ao HBsAg, os cinco casos positivos demonstram uma probabilidade de ocorrência menor que 1 x 10³ (p=0,059).

**Figura 1:** Fluxograma da testagem para o HBsAg e o anti-HCV segundo estrato socioeconômico em Recife (PE), Brasil, 2019.

A ocorrência das hepatites virais B e C, de acordo com sexo e faixas etárias, com seus respectivos intervalos de confiança, são apresentadas na Tabela 2. A maioria dos positivos para o HBsAg ou anti-HCV era de cor preta ou parda (6/7), tinha nível de escolaridade até o ensino médio (6/7), tinha um companheiro (5/7) e era do baixo estrato socioeconômico (5/7). Nenhum dos sete positivos referiu passado de transfusão de sangue ou derivados.

**Tabela 2:** Ocorrência da infecção pelo vírus da hepatite viral B e C de acordo com sexo e faixas etárias. Recife (PE), Brasil, 2019.

	Examinados	Hepatite B		Hepatite C	
		HBsAg		Anti-HCV	
		Pos	Prev (IC95%)	Pos	Prev (IC95%)
Todos	2.070	5	0,24 (0,04–0,50)	2	0,10 (0,00–0,24)
Sexo					
Feminino	1.212	3	0,27 (0,00–0,57)	1	0,10 (0,00–0,29)
Masculino	858	2	0,19 (0,00–0,66)	1	0,10 (0,00–0,30)
Grupo etário (em anos)					
≤40	1.173	4	0,41 (0,02–0,79)	1	0,10 (0,00–0,30)
>40	897	1	0,10 (0,00–0,29)	1	0,10 (0,00–0,29)

Pos: positivo; Prev: prevalência; IC95%: intervalo de confiança de 95%.

Os resultados da pesquisa do HBeAg e do HBV-DNA dos cinco casos identificados são apresentados na Tabela 3. Entre os dois casos anti-HCV positivo, um apresentou a pesquisa do HCV-RNA indetectável (referia tratamento antiviral prévio), e no outro a amostra biológica foi insuficiente para a pesquisa do RNA viral.

**Tabela 3:** Características demográficas e resultados do HBeAg e carga viral dos cinco pacientes com HBsAg positivo. Recife (PE), Brasil, 2019.

Caso	Sexo	Idade (anos)	Estrato socioeconômico	HBV-DNA		HBeAg
				UI/mL	Log	
1	F	39	Baixo	2.458	3.39	neg
2	M	38	Baixo	65	1.82	neg
3	M	36	Baixo	230.303.427	8.36	pos
4	F	56	Alto	8.008.840	6.90	pos
5*	F	38	Baixo	12	1.08	pos

* Em tratamento antiviral. F: feminino; M: masculino; Log: logaritmo; pos: positivo; neg: negativo.

## DISCUSSÃO

A ocorrência de HBsAg e de anti-HCV neste estudo foi menor do que a descrita em outros estudos brasileiros de base populacional^13,23^. Observou-se que a maior parte dos positivos residia no estrato socioeconômico baixo, cuja renda mensal do chefe da família era menor do que dois salários mínimos.

O primeiro inquérito de base populacional sobre a prevalência das hepatites virais A, B e C, em âmbito nacional, foi conduzido entre os anos de 2005 e 2009, quando foi verificado uma prevalência global pelo HBV menor que 1% e pelo HCV de 1,38% no conjunto das capitais de cada macrorregião do país e no Distrito Federal^13,14^. Na região Nordeste, particularmente, foi observado prevalência total do HBV^13^ de 5,5%, aproximadamente 20 vezes maior do que o observado no presente estudo (0,24%), e uma prevalência do HCV^14^ de 0,68%, bem próximo, porém um pouco maior do que foi verificado na nossa população (0,10%).

Outros estudos de prevalência das hepatites virais de base populacional foram conduzidos no Brasil. Estudo realizado em uma grande metrópole da região Sudeste, em 1996, verificou prevalência do HBsAg de 1,04% e do anti-HCV de 1,42%^23^. Posteriormente, em 1999, outro estudo de base populacional, realizado em uma pequena cidade da região semiárida da Bahia, constatou prevalência do HBsAg de 2,6% e do anti-HCV de 0,4%^24^. Passados 14 anos do último inquérito nacional^13,14^, os nossos resultados, avaliando 2.070 indivíduos da capital pernambucana, evidenciam uma redução na ocorrência das hepatites virais B e C nestes últimos anos. Certamente, tal fato se deveu à introdução da vacinação contra o HBV no calendário vacinal nacional e a melhoria da assistência aos casos, com a oferta de terapêuticas antivirais, a exemplo dos análogos nucleosídeos/nucleotídeos e antivirais de ação direta contra o HBV e o HCV, respectivamente^3,25^. Essas medidas contribuíram de forma substancial para redução da ocorrência dessas infecções, mormente nos países industrializados.

Corroborando resultados de estudos de prevalência nacionais e internacionais, que têm evidenciado a associação de fatores socioeconômicos, como o baixo nível de escolaridade e a baixa renda familiar^8,26-28^, a maior parte dos casos foi identificada no estrato socioeconômico baixo e tinha menor nível de escolaridade. Estimativas recentes mostraram que a maioria das infecções pelo HBV e pelo HCV estão distribuídos pelos países com precárias condições socioeconômicas^5^, onde o acesso à educação e à saúde, incluindo vacinas e medicamentos, são muito limitados, propiciando a persistência da transmissão.

Neste estudo, não se observou diferença estatisticamente significante entre a ocorrência de ambos os marcadores e a idade dos participantes, corroborando com estudos anteriores^13,14^. No que se refere ao sexo, as diferenças também foram semelhantes em relação a ocorrência dos marcadores virais B e C. Os nossos resultados corroboram com o estudo conduzido em todas as macrorregiões brasileiras, entre os anos de 2005 e 2009, quando foram pesquisados anticorpos da hepatite C na população^14^, mas divergem do estudo realizado em três regiões do Brasil, entre os anos de 2004 e 2005, que constatou uma associação positiva entre o sexo masculino e a infecção pelo HBV em todas as regiões estudadas^13^.

Vale destacar que, entre os cinco pacientes com HBsAg positivo, dois apresentavam viremias bastante elevadas e, em ambos, o HBeAg também resultou positivo. Todos os pacientes com marcadores positivos foram orientados a procurar serviços médicos de referência, inclusive o Hospital das Clínicas da Universidade Federal de Pernambuco.

O rastreamento de marcadores de infecção para o HBV e o HCV em uma soroteca proveniente de uma amostra populacional, cujo tamanho foi calculado para estimar a soroprevalência de arboviroses, constitui uma limitação deste estudo. Dada a disponibilidade da soroteca constituída com base em uma amostra aleatória da população geral, saudável *a priori*, não nos parece existir vieses que comprometam a validade do estudo.

Ressalta-se que estudos de prevalência envolvendo hepatites virais B e C em indivíduos saudáveis são raros, antigos e apresentam amostra menor, quando comparado ao nosso, com muito raras exceções. Por exemplo, estudo realizado no final da década de 1990 para avaliação da prevalência das hepatites virais A, B, C e E, na cidade de São Paulo, com população, na época, estimada em 9,6 milhões de habitantes, incluiu aleatoriamente 1.059 amostras de soros^23^. Em geral, os estudos mais recentes, com maior prevalência desses marcadores, foram conduzidos em grupos específicos da população, que têm maior risco de infecção, a exemplo dos profissionais do sexo^29^, dentre outros^15,16^.

A exclusão de indivíduos com mais de 65 anos constituiu outra limitação, considerando a relação da infecção pelo HCV com o aumento da idade^30^. No entanto não se observou na ocorrência de ambos os marcadores diferenças estatisticamente significantes quanto à idade.

Em síntese, os resultados deste estudo evidenciaram uma redução da ocorrência das hepatites virais B e C em um grande centro urbano da região Nordeste, que, de certo modo, refletem a situação atual da região. Ao mesmo tempo, os nossos resultados alertam para a necessidade de intensificação da vigilância epidemiológica e aprimoramento da rede assistencial dirigidas aos casos de HBV e HCV na população com menor condição socioeconômica. Após o diagnóstico nosológico, os indivíduos devem ser encaminhados para reavaliação e possível tratamento, acessível nos serviços de referência, para que se alcance a eliminação dessas viroses em nosso país até 2030, como foi proposto pela OMS^11^.
